# Efficacy and safety of acupuncture for chronic spontaneous urticaria: a systematic review and meta-analysis

**DOI:** 10.3389/fmed.2025.1648375

**Published:** 2026-01-28

**Authors:** Yaling Tong, Rong Jin, Yunfeng Yu, Shumin Xia, Zuqiang Wu, Xuan Su

**Affiliations:** 1 The Sixth Affiliated Hospital of South China University of Technology (Nanhai District People’s Hospital of Foshan), Foshan, Guangdong, China; 2 School of Traditional Chinese Medicine, Hunan University of Chinese Medicine, Changsha, Hunan, China; 3 Guangdong Integrated Traditional Chinese and Western Medicine Hospital, Foshan, Guangdong, China; 4 Foshan Nanhai Rulinwan Hospital of Integrated Traditional Chinese and Western Medicine, Foshan, Guangdong Province, China

**Keywords:** acupuncture, chronic spontaneous urticaria, complementary and alternative medicine, meta-analysis, systematic review

## Abstract

**Background:**

This systematic review and meta-analysis aimed to evaluate the efficacy and safety of acupuncture in the treatment of chronic spontaneous urticaria (CSU).

**Methods:**

Randomized controlled trials (RCTs) assessing acupuncture for CSU were retrieved from seven public databases up to March 2025. Meta-analyses were conducted for each outcome, and publication bias and the certainty of evidence were evaluated.

**Results:**

Eight RCTs involving 564 participants were included. Compared with antihistamines, acupuncture significantly improved the clinical effective rate (RR 1.19, 95% CI 1.10–1.29, *p* < 0.0001) and interferon-*γ* levels (MD 5.96, 95% CI 3.92–8.00, *p* < 0.00001). Acupuncture also significantly reduced the Dermatology Life Quality Index (DLQI) score (MD −3.39, 95% CI − 6.53 to −0.26, *p* = 0.03), immunoglobulin E levels (MD −13.95, 95% CI − 17.20 to −10.70, *p* < 0.00001), interleukin-4 levels (MD −6.24, 95% CI − 6.82 to −5.67, *p* < 0.00001), and the recurrence rate (RR 0.25, 95% CI 0.01–0.62, *p* = 0.003). The incidence of adverse events did not differ significantly between groups (RR 0.80, 95% CI 0.23–2.80, *p* = 0.73). Potential publication bias was observed for the clinical effective rate and DLQI.

**Conclusion:**

Compared with antihistamines, acupuncture may be a more effective alternative treatment for CSU. However, as the certainty of evidence was low, these findings require further validation.

## 1 Introduction

Chronic urticaria is a skin condition primarily characterized by recurrent episodes of wheals and itching, with individual lesions typically resolving within 24 h (1, 2). The disease often follows a prolonged and relapsing course, posing significant challenges for long-term management (2). Epidemiological investigations have shown that the point prevalence of chronic urticaria is approximately 0.7% (3). Notably, a nationwide cross-sectional study estimated that the point prevalence of chronic urticaria in China is as high as 2.6% (4). Chronic spontaneous urticaria (CSU) is the most common subtype, with disease duration ranging widely from several months to several years (5, 6). These clinical characteristics contribute to treatment complexity, and more than half of the patients experience psychiatric comorbidities such as sleep disturbances and anxiety, further compromising quality of life (7, 8). Currently, antihistamines such as loratadine and cetirizine are recommended as first-line pharmacological therapy (9, 10). When symptom control is inadequate, treatment may be escalated by combining multiple antihistamines or adding corticosteroids (11). Although these medications can relieve itching and other symptoms, their overall efficacy remains limited, and relapse frequently occurs once treatment is discontinued (12–14). Consequently, there is a growing need for safe and effective complementary treatment strategies to address the limitations of conventional therapies.

Acupuncture, a traditional therapy rooted in Traditional Chinese Medicine (TCM), exerts its therapeutic effects by stimulating specific acupoints on the body (15). Recent clinical research suggests that acupuncture may modulate the immune system through multiple pathways, exerting broad regulatory effects within the body’s complex immune network (16). It can suppress mast cell degranulation, reduce the release of inflammatory mediators, and thereby alleviate local inflammation and clinical symptoms (17). In addition, acupuncture appears to downregulate autoantibody levels, modulate cytokine profiles, inhibit immune cell recruitment to affected tissues, and reduce inflammatory infiltration, ultimately supporting tissue repair and immune homeostasis (18–20). Preliminary clinical studies have shown that acupuncture can reduce symptoms and relapse rates in patients with CSU, highlighting its potential as a complementary therapy (21).

A recent meta-analysis reported that acupuncture significantly improved clinical symptoms, quality of life, and serum biomarker levels in patients with CSU, supporting its use as a complementary treatment (22). However, this analysis combined trials of acupuncture alone with those of acupuncture plus antihistamines, introducing clinical heterogeneity and potential confounding, which may have reduced the precision and interpretability of the pooled estimates. Another meta-analysis found that acupuncture combined with antihistamines resulted in greater improvements in the Urticaria Activity Score over 7 days (UAS7) and Dermatology Life Quality Index (DLQI) compared with antihistamines alone (23). While this analysis supports the value of acupuncture as a supplement to standard therapy, it does not address whether acupuncture can serve as an alternative to antihistamines.

Although previous reviews have demonstrated the value of acupuncture as a complementary treatment, the feasibility of acupuncture as an alternative therapeutic strategy to antihistamines remains unclear. Therefore, this study aims to conduct a systematic review and meta-analysis in which the intervention consists of acupuncture alone or acupuncture combined with an antihistamine placebo, while the control comprises antihistamines or antihistamines combined with sham acupuncture. This design enables a rigorous assessment of the efficacy and safety of acupuncture as a potential alternative therapeutic option for CSU.

## 2 Materials and methods

### 2.1 Literature retrieval

A comprehensive literature search was conducted in CNKI, VIP Database, Wanfang, Embase, PubMed, Cochrane Library, and Web of Science to identify studies evaluating acupuncture for the treatment of CSU. The search strategy was applied to the Title/Abstract fields using the following terms: (Acupuncture OR Pharmacopuncture) AND (Urticaria OR Urticarias). The search covered all available records up to 31 March 2025, with no language restrictions. In addition, gray literature from clinical trial registries and reference lists of relevant reviews was screened to ensure completeness.

### 2.2 Inclusion and exclusion criteria

The inclusion criteria were as follows: (1) Study design: randomized controlled trials (RCTs); (2) Participants: patients diagnosed with CSU based on internationally recognized or Chinese diagnostic criteria. All participants were antihistamine-naïve and had not undergone acupuncture prior to enrollment; (3) Intervention: the experimental group received either acupuncture alone or acupuncture combined with an antihistamine placebo. No additional antihistamines or other medications outside the study protocol were permitted; (4) Control: the control group received standard-dose antihistamines or standard-dose antihistamines combined with sham acupuncture; (5) Outcomes: the primary outcome was the clinical effective rate, defined as the proportion of patients whose symptoms, such as wheals and pruritus, improved after treatment. Secondary outcomes included the DLQI, immunoglobulin E (IgE) levels, interleukin-4 (IL-4) levels, interferon-*γ* (IFN-γ) levels, and recurrence rate. Immunological biomarkers (IgE, IL-4, and IFN-γ) were analyzed using the absolute values reported in the included trials. A majority of studies provided mean (± SD) baseline and/or post-treatment absolute concentrations but did not report per-participant or per-group percentage change data. Assay methods and reporting units were comparable across studies, supporting the synthesis of absolute values in the meta-analysis. Safety outcomes were evaluated based on the incidence of adverse events.

The exclusion criteria were as follows: (1) duplicate publications or overlapping datasets, (2) studies involving pediatric populations, and (3) studies with inaccessible or incomplete outcome data.

### 2.3 Data extraction and quality assessment

A two-step screening process was used to identify eligible studies. First, duplicates and clearly irrelevant records were removed based on the inclusion and exclusion criteria. Second, the full texts of potentially eligible articles were reviewed to confirm final inclusion. From each included study, the following data were extracted: first author, publication year, participant characteristics, sample size, intervention details, treatment duration, and outcome measures.

Risk of bias was assessed using the Cochrane Risk of Bias tool, which evaluates seven domains: random sequence generation, allocation concealment, blinding of participants and personnel, blinding of outcome assessment, incomplete outcome data, selective reporting, and other potential sources of bias. Two reviewers independently performed data extraction and risk-of-bias assessment, with disagreements resolved through discussion with a third reviewer.

### 2.4 Statistical analysis

Meta-analyses were performed using Review Manager version 5.3. For dichotomous outcomes (clinical effective rate, recurrence rate, and adverse event rate), risk ratios (RRs) with 95% confidence intervals (CIs) were calculated. For continuous outcomes (DLQI score, IgE, IL-4, and IFN-γ levels), mean differences (MDs) with 95% CIs were calculated. Heterogeneity was assessed using the I^2^ statistic. An I^2^ value of <50% indicated low heterogeneity, in which case a fixed-effect model was applied; otherwise, a random-effects model was used. A *p*-value of < 0.05 was considered statistically significant.

Subgroup analyses were performed to explore the influence of acupuncture frequency and treatment duration on the primary outcome. Publication bias was assessed using funnel plots generated in Review Manager version 5.3, with symmetrical distribution interpreted as the absence of significant bias. Finally, the certainty of the evidence for each outcome was evaluated using the Grading of Recommendations Assessment, Development and Evaluation (GRADE) framework, considering five domains: risk of bias, inconsistency, indirectness, imprecision, and publication bias.

## 3 Results

### 3.1 Literature retrieval

**Figure 1:**
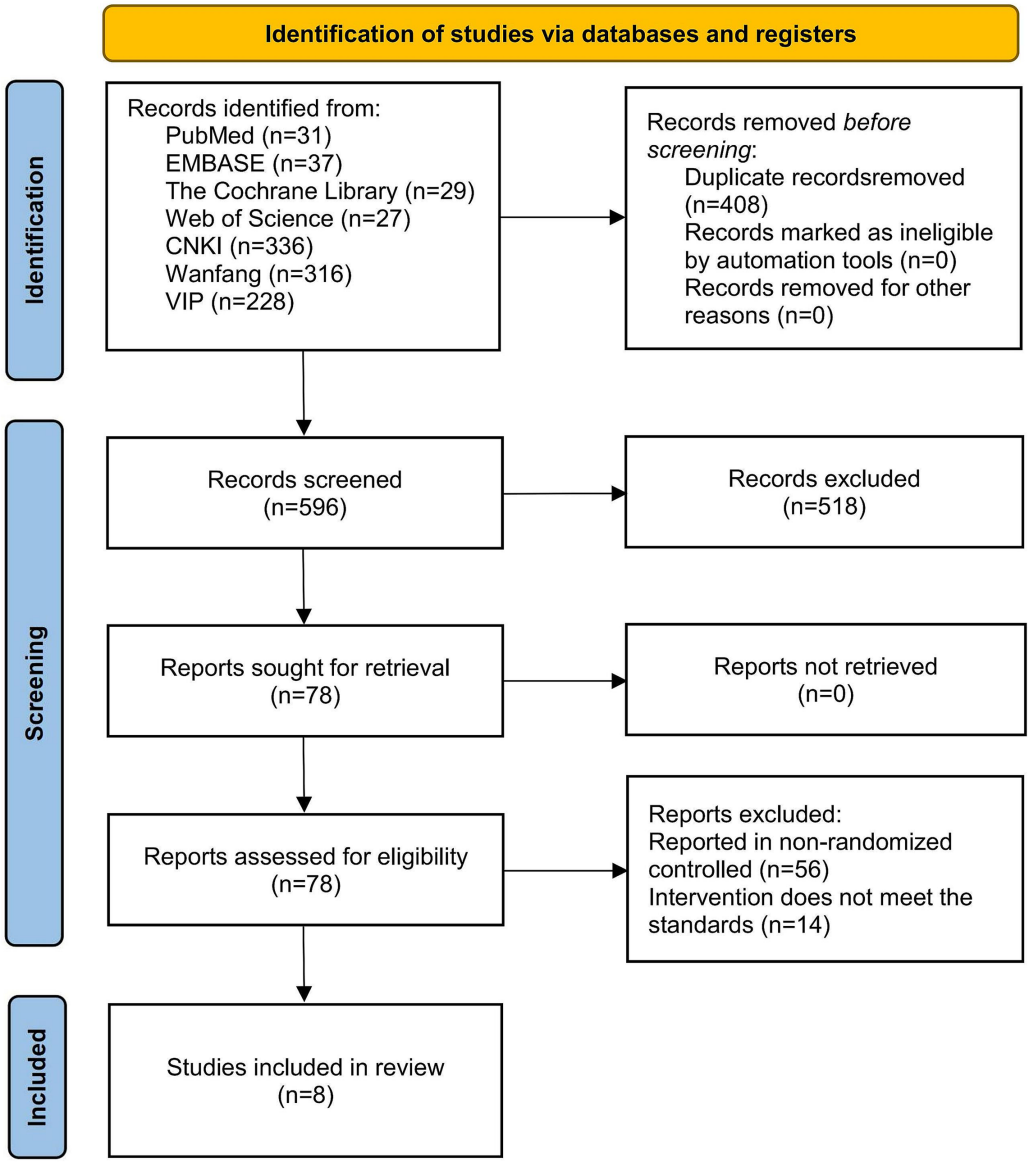
Flowchart of literature screening.

A total of 1,004 articles were initially identified from the 7 databases. After removing 408 duplicates, 596 articles remained. Screening of titles and abstracts led to the exclusion of 518 articles due to irrelevance. Full-text review of the remaining 78 articles resulted in the exclusion of 56 studies for non-randomized designs and 14 studies for interventions that did not meet the inclusion criteria. Ultimately, eight RCTs (24–31) were included in this systematic review and meta-analysis. The detailed screening process is illustrated in Figure 1.

### 3.2 Basic characteristics of the included studies

The eight included studies (24–31) were published between 2005 and 2022, and all originated from research institutions in China. The total sample size comprised 564 patients, with 283 in the acupuncture group and 281 in the antihistamine group. All participants were Chinese, with a mean age of 39.37 years, and 42.7% were male. Detailed characteristics of the included studies are presented in Table 1.

**Table 1:** Basic characteristics of the included studies.

Study	Country	Sample (E/C)	Male (%)	Age (years)	Disease duration (years)	Intervention	Comparison	Treatment duration (weeks)
Chen and Guo (24)	China	31/30	38.7	33.2	1.5	Acupuncture five times a week	Cetirizine 10 mg qd	4
Dai (25)	China	30/30	48.3	42.9	1.0	Acupuncture three times a week	Loratadine 10 mg qd	3
Gu et al. (26)	China	45/45	38.9	41.3	2.0	Acupuncture five times a week	Loratadine 10 mg qd	6
Wang et al. (27)	China	29/28	19.3	43.6	1.0	Acupuncture three times a week	Loratadine 10 mg qd	6
Wang et al. (28)	China	35/35	/	/	/	Acupuncture three times a week	Cetirizine 10 mg qd	4
Wen (29)	China	30/30	45.0	38.8	3.0	Acupuncture six times a week	Loratadine 10 mg qd	2
Wu et al. (31)	China	53/53	49.1	42.0	2.0	Acupuncture five times a week	Loratadine 10 mg qd	8
Zhang (30)	China	30/30	45.0	43.3	/	Acupuncture seven times a week	Fexofenadine 30 mg bid	4

### 3.3 Risk of bias assessment

**Figure 2:**
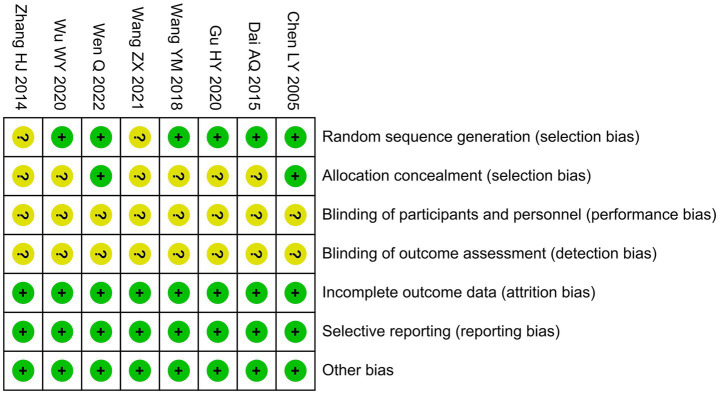
Risk of bias summary.

For random sequence generation, six studies clearly described appropriate randomization methods and were rated as having a low risk of bias. The remaining two studies did not provide sufficient details and were assessed as having an unclear risk. In terms of allocation concealment, only two studies reported specific measures, while the remaining studies did not provide relevant information, resulting in an unclear risk of bias. Regarding blinding of participants and personnel, none of the studies reported implementing blinding procedures. For blinding of outcome assessment, the primary outcomes of all included studies, such as the clinical effective rate and the DLQI, are inherently subjective and susceptible to assessor influence. Since none of the studies specified whether outcome assessors were blinded, the risk of detection bias for these subjective outcomes was considered unclear. In terms of incomplete outcome data, all studies reported complete outcome data without major loss to follow-up and were judged to have a low risk of bias. Similarly, no selective reporting was identified, and reporting bias was considered low risk across studies. No other obvious sources of bias, such as baseline imbalances or protocol deviations, were detected. A summary of the risk of bias assessment is shown in Figure 2.

### 3.4 Meta-analysis

#### 3.4.1 Clinical effective rate

**Figure 3:**
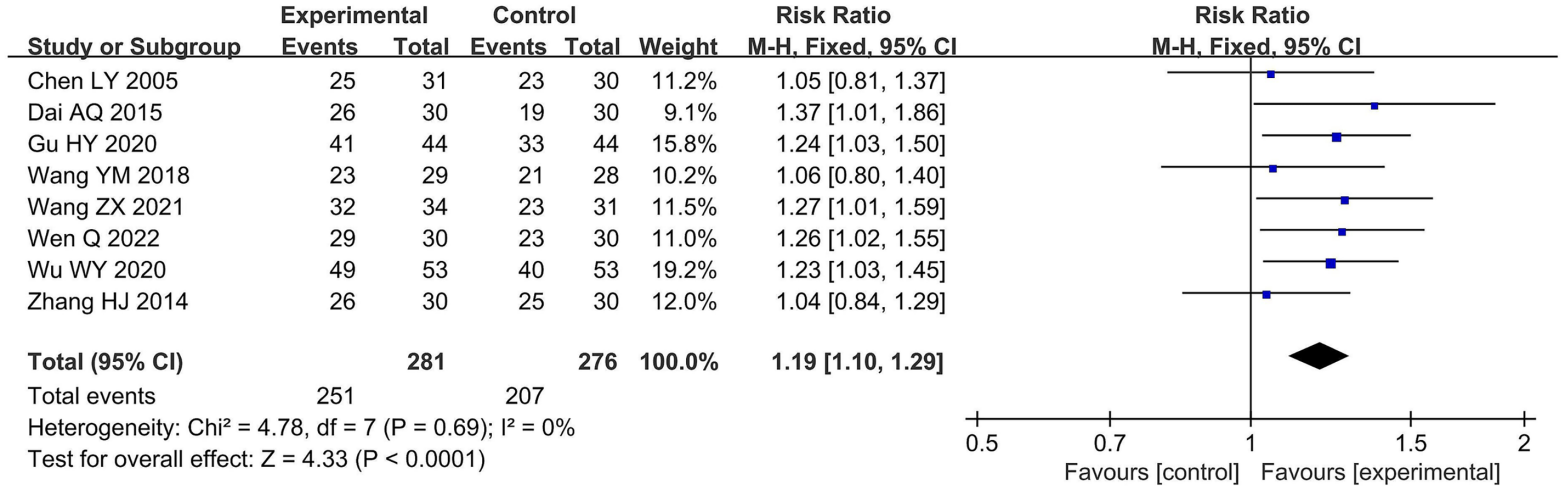
Forest plots of the meta-analysis on the clinical effective rate.

Eight studies involving a total of 557 patients reported data on the clinical effective rate. The meta-analysis showed that acupuncture significantly improved the clinical effective rate compared with antihistamine treatment (RR 1.19, 95% CI 1.10–1.29, *p* < 0.0001, I^2^ = 0%), as shown in Figure 3.

#### 3.4.2 DLQI

**Figure 4:**
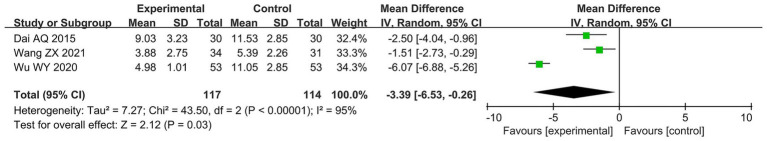
Forest plots of the meta-analysis of the Dermatology Life Quality Index (DLQI).

Three studies with a combined total of 231 patients assessed changes in DLQI scores. The results showed that acupuncture significantly reduced DLQI scores compared with antihistamine treatment (MD −3.39, 95% CI −6.53 to −0.26, *p* = 0.03, I^2^ = 95%), as shown in Figure 4.

#### 3.4.3 IgE, IL-4, and IFN-γ levels

**Figure 5:**
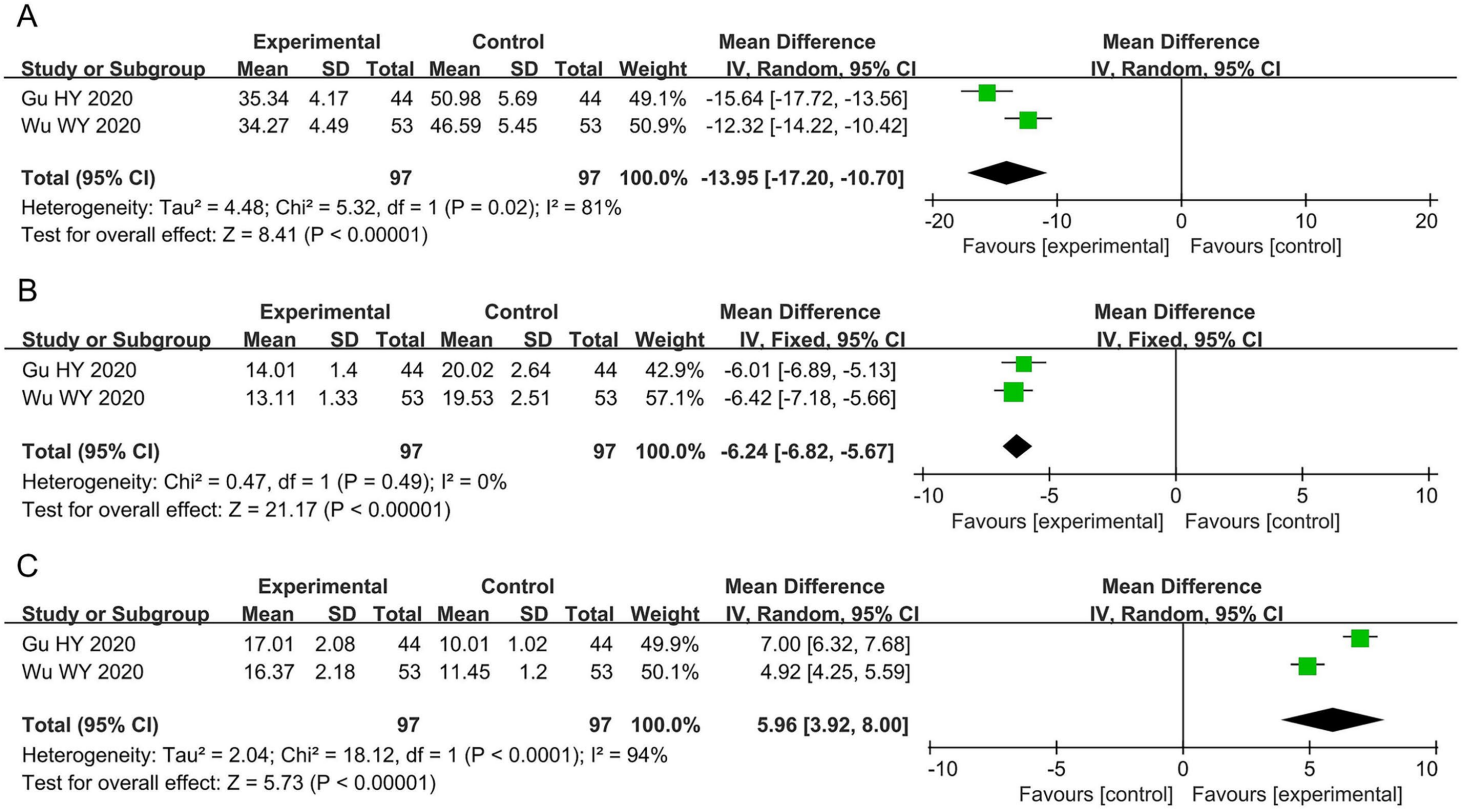
Forest plots of the meta-analysis of the levels of IgE, IL-4, and IFN-γ. **(A)** IgE, **(B)** IL-4, and **(C)** IFN-γ. IgE, immunoglobulin E; IL-4, interleukin-4; IFN-γ, interferon-γ.

Two studies involving 194 patients assessed IgE, IL-4, and IFN-γ levels. The results showed that, compared with antihistamines, acupuncture significantly decreased IgE levels (MD −13.95, 95% CI −17.20 to −10.70, *p* < 0.00001, I^2^ = 81%) and IL-4 levels (MD −6.24, 95% CI −6.82 to −5.67, *p* < 0.00001, I^2^ = 0%), while increasing IFN-γ levels (MD 5.96, 95% CI 3.92–8.00, *p* < 0.00001, I^2^ = 94%), as shown in Figure 5.

#### 3.4.4 Recurrence rate

**Figure 6:**
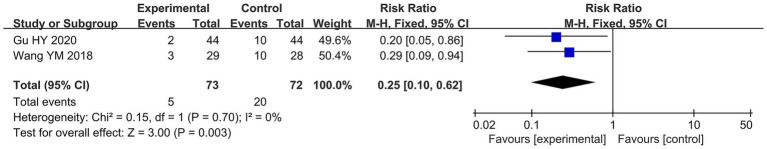
Forest plots of the meta-analysis on the recurrence rate.

Two studies with 155 patients reported recurrence rates. The findings indicated that acupuncture significantly reduced the recurrence rate of CSU compared with antihistamines (RR 0.25, 95% CI 0.10–0.62, *p* = 0.003, I^2^ = 0%), as shown in Figure 6.

#### 3.4.5 Safety outcomes

Meta-analyses of safety outcomes revealed that the incidence of adverse events was 3.88% (4/103) in the acupuncture group and 4.90% (5/102) in the antihistamine group. The difference between the two groups was not statistically significant (RR 0.80, 95% CI 0.23–2.80, *p* = 0.73, I^2^ = 0%). Specifically, no significant differences were observed in the incidence of headache (RR 0.06, 95% CI 0.00–0.98, *p* = 0.05, I^2^ = 0%), dizziness (RR 0.54, 95% CI 0.02–13.93, *p* = 0.71, I^2^ = 0%), nausea (RR 0.33, 95% CI 0.04–3.13, *p* = 0.34, I^2^ = 0%), blisters (RR 5.00, 95% CI 0.25–99.95, *p* = 0.29, I^2^ = 0%), ecchymoma (RR 5.00, 95% CI 0.25–99.95, *p* = 0.29, I^2^ = 0%), or stagnant needle (RR 3.00, 95% CI 0.13–70.83, *p* = 0.50, I^2^ = 0%), as summarized in Table 2.

**Table 2:** Meta-analysis of safety outcomes.

Outcome	Experimental	Control	I^2^	RR (95%CI)	*p-*value
Adverse event rate	4/103	5/102	0	0.80 (0.23, 2.80)	0.73
Headache	0/31	8/31	0	0.06 (0.00, 0.98)	0.05
Dizziness	1/74	4/74	0	0.54 (0.02, 13.93)	0.71
Nausea	0/74	2/74	0	0.33 (0.04, 3.13)	0.34
Blister	2/30	0/30	0	5.00 (0.25, 99.95)	0.29
Ecchymoma	2/30	0/30	0	5.00 (0.25, 99.95)	0.29
Stagnant needle	1/30	0/30	0	3.00 (0.13, 70.83)	0.50

### 3.5 Subgroup analysis

Subgroup analyses were conducted using the clinical effective rate as the outcome to explore the influence of acupuncture frequency and treatment duration on the meta-analysis results. Regarding acupuncture frequency, acupuncture performed three times per week (MD 1.23, 95% CI 1.05–1.43, *p* = 0.009), five times per week (MD 1.19, 95% CI 1.06–1.33, *p* = 0.003), and six times per week (MD 1.37, 95% CI 1.14–1.65, *p* = 0.0009) significantly increased the clinical effective rate, whereas seven times per week did not show a significant benefit (MD 1.04, 95% CI 0.84–1.29, *p* = 0.72). Regarding treatment duration, acupuncture administered for ≤ 4 weeks (MD 1.23, 95% CI 1.11–1.37, *p* < 0.0001) and > 4 weeks (MD 1.19, 95% CI 1.06–1.34, *p* = 0.003) both improved the clinical effective rate, as shown in Table 3.

**Table 3:** Subgroup analysis of the influence of acupuncture frequency and treatment duration on the clinical effectiveness rate.

Subject	Subgroup	I^2^	RR (95%CI)	*p-*value
Acupuncture frequency	Three times a week	0	1.23 (1.05, 1.43)	0.009
	Five times a week	0	1.19 (1.06, 1.33)	0.003
	Six times a week	0	1.37 (1.14, 1.65)	0.0009
	Seven times a week	0	1.04 (0.84, 1.29)	0.72
Treatment duration	≤ 4 weeks	15	1.23 (1.11, 1.37)	< 0.0001
	> 4 weeks	0	1.19 (1.06, 1.34)	0.003

### 3.6 Publication bias

**Figure 7:**
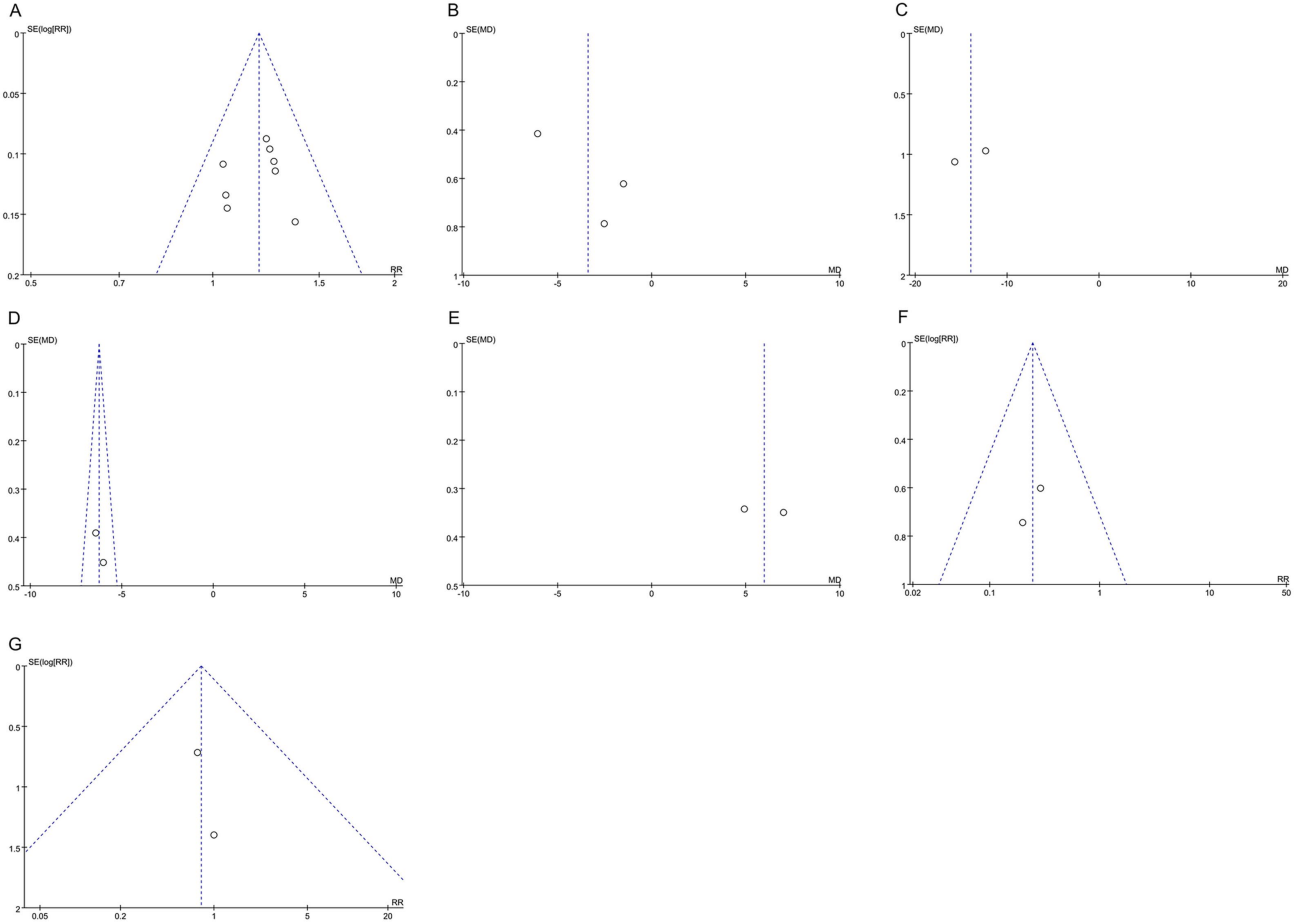
Funnel plots of publication bias. **(A)** clinical effective rate, **(B)** DLQI, **(C)** IgE, **(D)** IL-4, **(E)** IFN-γ, **(F)** recurrence rate, and **(G)** adverse event rate. DLQI, Dermatology Life Quality Index; IgE, immunoglobulin E; IL-4, interleukin-4; and IFN-γ, interferon-γ.

Funnel plots showed that the scatter distributions of the clinical effective rate, IgE, IL-4, IFN-γ, recurrence rate, and adverse event rate were symmetrical, indicating no publication bias. However, the scatter distributions of DLQI were asymmetrical, indicating potential publication bias, as shown in Figure 7.

### 3.7 Certainty of evidence

The GRADE system showed that the certainty of evidence was low for the clinical effective rate, IL-4, IFN-γ, recurrence rate, and adverse event rate, while the certainty of evidence for DLQI and IgE was very low, as shown in Table 4.

**Table 4:** Certainty of evidence.

Outcomes	Limitation	Inconsistency	Indirectness	Imprecision	Publication bias	Certainty of evidence
Clinical effective rate	Serious	Not serious	Not serious	Not serious	Serious	Low
DLQI	Serious	Very serious	Not serious	Serious	Serious	Very Low
IgE	Serious	Very serious	Not serious	Serious	Not serious	Very Low
IL-4	Serious	Not serious	Not serious	Serious	Not serious	Low
IFN-γ	Serious	Very serious	Not serious	Serious	Not serious	Low
Recurrence rate	Serious	Not serious	Not serious	Serious	Not serious	Low
Adverse event rate	Serious	Not serious	Not serious	Serious	Not serious	Low

DLQI, Dermatology Life Quality Index; IgE, immunoglobulin E; IL-4, interleukin-4; IFN-γ, interferon-γ.

## 4 Discussion

### 4.1 Research background and significance

CSU is a common dermatological condition characterized by recurrent episodes and a prolonged disease course. Antihistamines and corticosteroids are the primary treatment options; however, concerns remain regarding their long-term safety and efficacy (10). In addition, the potential adverse effects associated with these medications may lead to poor adherence for some patients (7). In contrast, acupuncture, a traditional Chinese medicine therapy, provides a non-pharmacological alternative for patients with CSU (11). Previous studies have suggested that acupuncture may reduce the risk of drug-related adverse events, thereby improving patient compliance and satisfaction (11). As a safe, externally administered therapy, acupuncture is often more acceptable to patients and may contribute to better clinical outcomes. To our knowledge, this is the first meta-analysis to explore the clinical value of acupuncture as an alternative to antihistamines in the treatment of CSU.

### 4.2 Efficacy of acupuncture treatment

The clinical effective rate reflects the proportion of patients whose symptoms, such as wheals and pruritus, improve after treatment, thereby indicating its therapeutic effectiveness for CSU. Our findings show that, compared with antihistamines, acupuncture resulted in a significantly higher clinical effective rate, underscoring its advantages in alleviating symptoms and improving clinical signs of CSU.

The DLQI is commonly used to evaluate patients’ quality of life and psychological well-being. It encompasses physical symptoms as well as emotional state, daily living activities, clothing choices, social interactions, physical exercise, work performance, sexual life, and treatment burden (31). Our study demonstrated that acupuncture significantly reduced DLQI scores compared with antihistamines, suggesting that acupuncture may offer additional benefits in enhancing the overall quality of life of patients with CSU.

Biomarkers such as IgE, IL-4, and IFN-γ are frequently used to assess allergic status and immune function in CSU. IgE is a cytophilic antibody central to type I hypersensitivity, and its mast-cell-mediated activation and subsequent release of inflammatory mediators play a key role in CSU pathogenesis (32). IL-4 promotes humoral immunity by regulating IgE production and is a pivotal cytokine in allergic responses (33). IFN-γ, primarily produced by Th1 cells, suppresses IgE secretion by B cells and thus mitigates type I hypersensitivity reactions (34). Our results show that acupuncture significantly reduced IgE and IL-4 levels and increased IFN-γ levels compared with antihistamines. These findings suggest that acupuncture may alleviate hypersensitivity and inflammation by modulating immune responses, representing a potential therapeutic mechanism in CSU treatment.

In summary, acupuncture improves clinical symptoms, enhances quality of life, and regulates immune and inflammatory responses in patients with CSU. These findings support the potential value of acupuncture as a complementary and alternative therapeutic option for CSU.

### 4.3 Safety of acupuncture treatment

Regarding safety outcomes, the meta-analysis revealed no statistically significant difference in the incidence of adverse events between the acupuncture and antihistamine groups (RR 0.80, 95% CI 0.23–2.80, *p* = 0.73), indicating that acupuncture has a safety profile comparable to that of antihistamines. Furthermore, no significant differences were observed between the two groups in the incidence of specific adverse events, including headache (RR 0.06, 95% CI 0.00–0.98, *p* = 0.05), dizziness (RR 0.54, 95% CI 0.02–13.93, *p* = 0.71), nausea (RR 0.33, 95% CI 0.04–3.13, *p* = 0.34), blisters (RR 5.00, 95% CI 0.25–99.95, *p* = 0.29), ecchymoma (RR 5.00, 95% CI 0.25–99.95, *p* = 0.29), and stagnant needle (RR 3.00, 95% CI 0.13–70.83, *p* = 0.50). All comparisons exhibited low heterogeneity, further enhancing the reliability of the pooled estimates. These findings indicate that acupuncture is generally safe and well tolerated, without an increased risk of adverse events compared with conventional antihistamine therapy.

Nevertheless, these safety results should be interpreted with caution. For one, the included studies had relatively small sample sizes, limiting statistical power and generalizability. For another, the wide confidence intervals for several adverse events reflect substantial uncertainty and potential variability in the risk estimates. Therefore, future large-scale, high-quality trials are needed to more precisely evaluate the safety profile of acupuncture and confirm these preliminary findings.

### 4.4 Mechanism of acupuncture treatment

Previous studies suggest that acupuncture may exert therapeutic effects on CSU through multiple mechanisms (35–37). First, acupuncture can modulate mast cell activation by regulating various signaling pathways, thereby inhibiting degranulation and the subsequent production and release of inflammatory mediators (38, 39). Second, acupuncture can restore immune balance by modulating the ratios of CD4^+^/CD8^+^ T cells, Th1/Th2 cells, and Th17/Treg cells (16), which may alleviate symptoms such as local edema and pruritus through multiple immunological pathways. Moreover, acupuncture may directly reduce serum IgE levels, regulate systemic cytokine profiles, and suppress the release of inflammatory mediators, including IL-4, IL-6, and TNF-α (40–42). In summary, the therapeutic effects of acupuncture in CSU appear to be mediated by the multidimensional modulation of the body’s complex immune network, targeting multiple immune pathways to relieve cutaneous symptoms and correct underlying immune dysregulation.

### 4.5 Limitations and prospects

Although this study followed PRISMA guidelines, several limitations should be acknowledged. First, the meta-analysis included only 564 participants, which may compromise the precision and statistical power of the results. Second, two of the included studies restricted the participants’ age to 20–65 years, potentially limiting the generalizability of the findings to other age groups. Third, all nine clinical trials exclusively enrolled Chinese participants, which limits the applicability of the findings to other ethnic populations, such as Europeans, Africans, and Latin Americans. Fourth, the treatment durations ranged from 4 to 8 weeks, indicating that the findings primarily reflect the short-term efficacy of acupuncture, with no long-term follow-up data available. Fifth, most included trials used subjective outcomes, such as the clinical effective rate and DLQI, as primary endpoints, yet none reported whether outcome assessors were blinded. The lack of assessor blinding may introduce detection bias, thereby reducing confidence in the observed treatment effects for these subjective outcomes.

In light of these limitations, future research should focus on (i) strengthening the evidence base through multicenter, stratified randomized controlled trials; (ii) conducting international studies in European, African, and Latin American populations to assess ethnic differences in treatment response; (iii) improving methodological rigor by clearly reporting blinding procedures, particularly for subjective clinical outcomes; and (iv) extending follow-up durations to more than 6 months to evaluate long-term efficacy and safety outcomes.

## 5 Conclusion

Compared with antihistamine therapy, acupuncture significantly improved clinical symptoms, enhanced quality of life, regulated key immunological biomarkers, reduced recurrence rates, and demonstrated a favorable safety profile in patients with CSU. However, further studies are needed to evaluate acupuncture’s clinical value for CSU and elucidate its underlying molecular mechanisms.

## Data Availability

The original contributions presented in the study are included in the article/Supplementary material, further inquiries can be directed to the corresponding author.
